# Prevalence of risk factors for dementia in middle- and older- aged people registered in Primary Health Care

**DOI:** 10.1590/1980-57642021dn15-020012

**Published:** 2021

**Authors:** Bruna Moretti Luchesi, Beatriz Rodrigues de Souza Melo, Priscila Balderrama, Aline Cristina Martins Gratão, Marcos Hortes Nisihara Chagas, Sofia Cristina Iost Pavarini, Tatiana Carvalho Reis Martins

**Affiliations:** 1 Undergraduate Medical School, Universidade Federal de Mato Grosso do Sul, Campus de Três Lagoas - Três Lagoas, MS, Brazil.; 2 Graduate Program in Nursing, Universidade Federal de Mato Grosso do Sul, Campus de Três Lagoas - Três Lagoas, MS, Brazil.; 3 Graduate Program in Nursing, Universidade Federal de São Carlos - São Carlos, SP, Brazil.; 4 Graduate Program in Gerontology, Universidade Federal de São Carlos - São Carlos, SP, Brazil.; 5 Bairral Institute of Psychiatry - Itapira, SP, Brazil.

**Keywords:** aged, dementia, middle aged, primary prevention, Primary Health Care, risk factors, idoso, demência, pessoa de meia-idade, prevenção primária, Atenção Primária à Saúde, fatores de risco

## Abstract

It is important to assess the prevalence of risk factors for dementia to slow down the progression and evolution of the disease, and to support interventions and prevention programs.

**Objective:**

We aimed to evaluate the prevalence of these factors in individuals registered in Primary Health Care in Brazil and their relationship with sex and age group.

**Methods:**

This was a cross-sectional and quantitative study with n=300 individuals. We evaluated the prevalence of main risk factors (low education, hearing loss, high blood pressure, obesity, smoking, depression, physical inactivity, social isolation, and diabetes mellitus) and others (poor diet, alcohol use, head trauma, monolingualism, visual impairment, and sleep disorders) identified in the literature. Poisson regression was used, according to sex and age group (45-59 years/60+ years).

**Results:**

The main risk factors with the highest prevalence were physical inactivity (60.3%) and depressive symptoms and hypertension (56.7% each). Among the other factors, monolingualism (98.0%), visual impairment (84.7%), and irregular consumption of fruits (60.4%), and vegetables (53.5%) prevailed. No differences were identified between sexes. The regression analysis confirmed a significant difference for education and age group, with older individuals having a higher prevalence of low schooling.

**Conclusion:**

The results can guide interventions, especially in developing countries. Practice of physical activity and healthy eating should be the focus of these interventions as they can indirectly help in reducing the prevalence of other factors. Early identification, screening and adequate treatment of depressive symptoms, high blood pressure and visual impairment can also contribute to reducing the prevalence of dementia.

## RESUMO.

A identificação da prevalência de fatores de risco para demência é importante para reduzir a velocidade da progressão e evolução da doença, e para subsidiar programas de prevenção e intervenção.

**Objetivo:**

O objetivo foi avaliar a prevalência desses fatores em indivíduos cadastrados na Atenção Primária à Saúde (APS), relacionando com sexo e faixa etária.

**Métodos:**

Estudo transversal e quantitativo, com n=300 indivíduos acima de 45 anos. Avaliou-se a prevalência dos fatores de risco principais para demência (baixa escolaridade, perda auditiva, hipertensão arterial, obesidade, tabagismo, depressão, inatividade física, isolamento social e diabetes mellitus) e dos outros fatores (má alimentação, consumo de álcool, traumatismo craniano, unilinguismo, déficit visual e distúrbios do sono), identificados na literatura. Conduziu-se uma regressão de Poisson, por sexo e faixa etária (45-59 e 60+ anos).

**Resultados:**

Os fatores de risco principais com maior prevalência foram a inatividade física (60,3%), a sintomatologia depressiva e a hipertensão arterial (56,7% cada). Dentre os outros fatores, prevaleceram: unilinguismo (98,0%), déficit visual (84,7%), consumo irregular de frutas (60,4%) e de verduras ou legumes (53,5%). Não foram identificadas diferenças entre os sexos. Confirmou-se diferença significativa para a escolaridade e faixa etária, com os idosos apresentando maior prevalência de baixa escolaridade.

**Conclusão:**

Os resultados podem guiar intervenções, especialmente em países em desenvolvimento. A prática de atividades físicas e a alimentação devem ser o foco dessas intervenções, auxiliando indiretamente na redução da prevalência de outros fatores. A identificação precoce, o rastreamento e o tratamento adequado de sintomas depressivos, hipertensão arterial e déficit visual também são importantes.

## INTRODUCTION

As the world population ages, dementia is becoming an important public health problem.^1^ Dementia can be defined as a syndrome of multifactorial etiology, with the presence of cognitive deficit and gradual loss of ability to perform activities of daily living and social functioning, leading individuals to dependence and loss of autonomy.^2^ It is estimated that around 50 million people worldwide have dementia, with 60% living in low- and middle-income countries.^3^ Its enormous social and economic burdens threaten, along with other chronic diseases, the sustainability of health care systems.^4^ The costs of dementia are very high for the health system, the family and the community. One of the biggest challenges related to dementia syndromes is the control of risk factors to slow down the evolution and progression of the disease.^2^

Accordingly, in 2017, “The Lancet Commission on Dementia Prevention, Intervention, and Care” established the risk and protection factors for dementia with the goal to consolidate the advances and the emerging knowledge about what we must do to prevent and manage this disease.^2^ It was identified that 35% of the factors are potentially modifiable, and the other 65% are genetic and environmental factors. The fraction of the modifiable factors attributable to the population was calculated, that is, the percentage reduction in new cases over a certain period of time, if a particular risk factor was completely eliminated.^2^ The authors considered studies mostly conducted in high-income countries.

The modifiable risk factors were divided into those that occur in childhood, middle age and old age. Low schooling (even primary education) in young age was responsible for 8%. Hearing loss (9%), high blood pressure (2%) and obesity (1%) were identified as relevant factors in middle age. In turn, smoking (5%), depression (4%), physical inactivity (3%), social isolation (2%) and diabetes mellitus (1%) were identified in old age.^2^ Some factors were not confirmed as risk factors, but there is evidence of their influence on the development of dementia, and they are referred to as “other factors”. They include poor diet, alcohol use, head trauma, monolingualism, visual impairment, and sleep disorders.^2^ In July 2020, the document was updated, and included the excessive alcohol consumption, traumatic brain injury, and air pollution as main risk factors. The percentage of potentially modifiable factors increased to 40%.^5^ In the present study, we evaluated the factors published in 2017, however; the new publication must be considered in further studies.

The same analysis of 2017 was later carried out in low- and middle- income countries,^6^ evaluating representative samples from India, China and six countries in Latin America (Cuba, Dominican Republic, Mexico, Peru, Puerto Rico and Venezuela). Modifiable risk factors for dementia accounted for 39.5% of the total in China, 41.2% in India and 55.8% in Latin America. Five factors were more prevalent than those previously identified:^2^ low education (10.8, 13.6 and 10.9% in China, India and Latin America, respectively), smoking (14.7, 6.4 and 5.5%), hypertension (6.4, 4.0 and 9.3%), obesity (5.6, 2.9 and 7.9%) and diabetes mellitus (1.6, 1.7 and 3.2%).^6^

A new study was carried out in Mozambique, Brazil and Portugal, countries that have a similar culture, but different income patterns. Seven modifiable risk factors were assessed and found to be associated with dementia in 24.4% of the cases in Mozambique, 32.3% in Brazil, and 40.1% in Portugal.^7^ In Brazil, the prevalence of risk attributable to each factor was: physical inactivity (27.4%), low schooling (21.0%), smoking (8.1%), depression (4.7%), high blood pressure and obesity (4.3% each), and diabetes mellitus (2.8%). It was identified that if the prevalence of each factor decreased by 20.0% per decade, there would be a potential reduction of 16.2% in the prevalence of dementia by 2050.^7^

It should be noted that although these factors are evident in old age, they are important throughout the course of life, especially during middle age. The aim of this article was to assess the prevalence of risk factors for dementia in middle- and older-aged people registered in Primary Health Care (PHC) units and their relationship with sex and age group. The identification of these prevalence levels may support interventions and prevention programs in the future, which can be targeted specifically at men, women and age group, according to the results.

## METHODS

This was a cross-sectional and quantitative study carried out in PHC units in the municipality of Três Lagoas, MS, Brazil. According to the 2010 census (the last official report), the city had 101,791 habitants, of which 16.1% were between 45-59 years old and 9.9% were older adults (≥60 years). In 2018, the total population was estimated to be 119,465, and the city had a total of nine units and 14 PHC teams serving the Family Health Strategy (41.1% coverage).

The study population was adult (45-59 years) and older adult (≥60 years) individuals. The inclusion criteria were age of 45 years or older and registration in a PHC unit in the city. Users with mental illness or disorder or untreated systemic diseases that prevented participation were excluded.

The sample was determined according to the formula for estimating the proportion of adult and older adult individuals in finite populations. The significance level of 5% (alpha=0.05), a sampling error of 5% (e=0.05), an estimate of 50% (p=0.50) (whose value provides the largest sample size necessary for sample representativeness), and a finite population size of 200 individuals in each age group were used. A minimum sample of n=132 subjects was obtained in each group (132 adult and 132 older adult individuals). Throughout the collection, 147 adults and 153 older adults were evaluated, resulting in a sampling error of 4.2% for adults and 3.9% for older adults.

The selection of participants followed the recommendation of the PHC teams, which provided a list of individuals in the area covered by the unit who met the inclusion criteria. A total of 369 adult and older adult individuals were visited; nine of them were not found after two visits, one had changed address, and 59 refused to participate, resulting in a sample of 300 participants (n=147 adult and n=153 older adult individuals), with a response rate of 81.3%. A minimum of 25 people was respected in each of the nine units in the city, to ensure the participation of all areas covered.

Interviews were conducted at the home or at the facilities of the health unit, according to the availability of the participants, from November 2018 to June 2019. The interviews were individual and conducted by trained evaluators.

Sociodemographic data including sex, age, marital status, and personal and family income (in minimum wages) were collected.

To assess the main risk factors for dementia, we considered:

- Schooling: in number of years.
- Hypertension: self-reported (“Has any doctor ever told you that you have high blood pressure?”).
- Diabetes mellitus: self-reported (“Has any doctor ever told you that you have diabetes?”).
- Obesity: assessed based on the body mass index (BMI), which corresponds to the calculation of weight (kg)/height (m)^2^. Obesity was considered to be present in adult individuals when BMI≥30 kg/m^2^, and in older adult individuals with BMI≥27 kg/m^2^.^8^ Weight and height were measured in triplicate and the average of three measures was used.
- Hearing loss: assessed through question 19 of the Clinical-Functional Vulnerability Index-20 (IVCF-20) “Do you have hearing problems that can prevent you from carrying out any daily activity?”.^9^
- Smoking: self-reported (“Do you currently smoke?”).
- Social isolation: self-reported (“Do you consider yourself socially isolated?”).
- Depression: assessed through the Center for Epidemiological Studies - Depression (CES-D), validated in Brazil.^10^ The instrument has 20 items ranging from 0 to 3 points each that assess the frequency of depressive symptoms experienced in the week prior to the interview (never, rarely, most of the time, always). The final score ranges from zero to 60 points. For adults, the cut-off score for the presence of depressive symptoms is ≥16 points, and for the older adults, ≥12 points.
- Physical inactivity: assessed by the International Physical Activity Questionnaire (IPAQ) short version, validated in Brazil.^11^ Individuals who reported at least 75 minutes a week of vigorous physical activity or 150 minutes a week of moderate activity were considered active.^12^

As for the “other factors”, the following ones were considered:

- Head trauma: “Have you ever suffered any head trauma (loss of consciousness >1 hour)?”.
- Visual impairment: assessed by the question 18 of the IVCF-20 “Do you have vision problems that prevent you from carrying out any daily activity?”.^9^
-Sleep disorders: self-reported (“Do you have any sleep disorder?”).
- Monolingualism: self-reported (“How many languages are you fluent in?”).
- Alcohol use: self-reported (“Do you currently use alcohol?”).
- Food: according to the questionnaire used in population surveys in Brazil, using the items weekly frequency of consumption of beans, vegetables, fruits, soft drinks and sweets (ice cream, chocolates, cakes, cookies). Regular consumption was considered to be five or more days a week.^12^

Prevalence and respective 95% confidence intervals were estimated for each risk factor, according to sex and age group (45-59 years and 60 or more years). Proportions were compared using Pearson’s chi-square tests; and adjusted prevalence ratios were calculated by Poisson regression, considering values of p≤0.05 statistically significant. Analyses were performed using the Statistical Package for the Social Sciences version 25.0.

The project was approved by the Human Research Ethics Committee of the Federal University of Mato Grosso do Sul, and data collection started only after approval and after all participants read and signed an informed consent form.

## RESULTS

Table 1 shows the participants’ sociodemographic and health data. Most were female, older adults, had no partner, and had personal income of up to one minimum wage and family income of up to two minimum wages.

**Table 1.** Sociodemographic characterization of adults and older adults registered in Primary Health Care.

	%
Sex	
Female	65.7
Male	34.3
Age group	
45‒59 years	49.0
60 years or older	51.0
Marital status	
With partner	44.0
Without partner	56.0
Personal income	
Up to 1 minimum wage	51.3
More than one minimum wage	36.7
Not informed	12.0
Family income	
Up to 2 minimum wages	41.0
More than 2 minimum wages	34.3
Not informed	24.7

The prevalence of risk factors for dementia and the prevalence ratio in the general sample and by sex are shown in Table 2 and in the general sample and by age group in Table 3.

**Table 2.** Prevalence and prevalence ratio of risk factors for dementia in adults and older adults registered in Primary Health Care, according to sex.

	Total		Sex				PR (Female/Male) Adjusted analysis	
			Male		Female			
	%	95%CI	%	95%CI	%	95%CI	PR	95%CI
Main variables								
Schooling (up to 4 years)	44.0	38.5-49.7	51.5	38.7-58.4	40.1	33.2-47.0	0.96	0.79-1.16
Hypertension (yes)	56.7	51.0-62.0	59.2	50.0-69.0	55.3	48.0-62.0	0.95	0.78-1.15
Diabetes Mellitus (yes)	26.7	21.2-31.9	24.3	16.0-33.0	27.9	22.0-34.0	1.01	0.81-1.25
Obesity (yes)	47.4	41.7-53.0	43.7	33.9-53.4	49.5	42.4-56.5	1.02	0.84-1.25
Hearing loss (yes)	17.3	13.5-22.0	13.6	7.0-20.0	19.3	14.0-25.0	1.04	0.81-1.32
Smoking (yes)	18.7	14.7-23.5	21.4	13.0-29.0	17.3	12.0-23.0	0.97	0.75-1.25
Social isolation (yes) *	27.0	22.3-32.3	17.5	10.0-25.0	32.0	25.0-39.0	1.05	0.84-1.31
Depression (yes) *	56.7	51.0-62.1	45.6	36.0-55.0	62.4	56.0-69.0	1.09	0.88-1.35
Physical inactivity	60.3	54.7-65.7	60.8	51.0-70.0	61.3	32.0-46.0	1.01	0.83-1.21
Other factors								
Head trauma (yes)	9.7	6.8-13.5	7.8	3.0-13.0	10.7	6.0-15.0	1.02	0.76-1.38
Visual impairment	84.7	80.1-88.3	79.6	72.0-88.0	87.3	83.0-92.0	1.05	0.80-1.37
Sleep disorder*	39.7	34.3-45.3	23.3	15.0-32.0	48.2	41.0-55.0	1.11	0.91-1.34
Monolingualism	98.0	95.7-99.1	97.1	94.0-99.0	98.5	97.0-99.0	1.12	0.57-2.23
Alcohol use*	23.3	18.9-28.4	30.1	21.0-39.0	19.8	14.0-25.0	0.94	0.75-1.18
Food								
Beans (irregular consumption)*	30.5	25.4-35.9	22.5	14.3-30.8	34.7	28.0-41.4	1.08	0.88-1.31
Vegetables or greens (irregular consumption)	53.5	47.8-59.2	61.2	51.6-70.7	49.5	42.4-56.6	0.92	0.75-1.12
Fruits (irregular consumption)	60.4	54.7-65.9	62.1	52.6-71.7	59.5	52.5-66.4	0.99	0.82-1.21
Soft drinks (regular consumption)*	19.7	15.6-24.5	26.2	17.6-34.8	16.3	11.1-21.5	0.93	0.73-1.17
Sweets (regular consumption)	22.3	17.9-27.4	21.4	13.3-29.4	23.0	17.0-28.9	1.02	0.82-1.28

PR: prevalence ratio; reference category - male individuals. 95%CI: 95% confidence interval. *Significant difference (p≤0.05) according to the chi-square test.

**Table 3.** Prevalence and prevalence ratio of risk factors for dementia in adults and older adults registered in Primary Health Care, according to age group.

	Total		Age				PR (older adult/adult) Adjusted analysis	
			Adult		Older adult			
	%	95%CI	%	95%CI	%	95%CI	PR	95%CI
Main variables								
Schooling (up to 4 years)*	44.0	38.5-49.7	23.1	51.0-67.0	62.1	56.4-71.7	1.27	1.04-1.55
Hypertension (yes)*	56.7	51-62	40.8	33.0-49.0	71.9	65.0-79.0	1.16	0.94-1.44
Diabetes mellitus (yes)*	26.7	21.2-31.9	19.7	13.0-26.0	33.3	26.0-41.0	1.04	0.84-1.31
Obesity (yes)*	47.4	41.7-53.0	38.4	30.4-46.3	56.2	48.3-64.2	1.07	0.87-1.31
Hearing loss (yes)	17.3	13.5-22.0	13.6	8.0-19.0	20.9	14.0-27.0	1.05	0.81-1.35
Smoking (yes)	18.7	14.7-23.5	21.8	15.0-29.0	15.7	10.0-22.0	0.99	0.76-1.30
Social isolation (yes)	27.0	22.3-32.3	28.6	21.0-36.0	25.5	19.0-32.0	0.95	0.75-1.20
Depression (yes)	56.7	51.0-62.1	55.1	47.0-63.0	58.2	50.0-66.0	1.01	0.81-1.26
Physical inactivity	60.3	54.7-65.7	61.1	53.0-69.0	61.2	31.0-47.0	1.02	0.83-1.24
Other factors								
Head trauma (yes)	9.7	6.8-13.5	8.8	4.0-13.0	10.5	6.0-15.0	1.02	0.74-1.40
Visual impairment	84.7	80.1-88.3	86.4	81.0-92.0	83.0	77.0-89.0	0.94	0.72-1.35
Sleep disorder	39.7	34.3-45.3	40.1	32.0-48.0	39.2	31.0-47.0	0.97	0.79-1.19
Monolingualism	98.0	95.7-99.1	98.6	97.1-99.9	97.4	95.0-99.0	0.83	0.43-1.61
Alcohol use	23.3	18.9-28.4	26.5	19.0-34.0	20.3	14.0-27.0	0.99	0.78-1.27
Food								
Beans (irregular consumption)*	30.5	25.4-35.9	37.0	29.1-44.9	24.3	17.4-31.2	0.91	0.73-1.12
Vegetables or greens (irregular consumption)	53.5	47.8-59.2	54.1	45.9-62.3	52.9	44.9-60.9	0.99	0.81-1.22
Fruits (irregular consumption)	60.4	54.7-65.9	64.1	56.3-72.0	56.9	48.9-64.8	0.97	0.79-1.19
Soft drinks (regular consumption)*	19.7	15.6-24.5	26.0	18.8-33.2	13.7	8.2-19.2	0.93	0.72-1.29
Sweets (regular consumption)*	22.3	17.9-27.4	28.1	20.7-35.5	17.0	11.0-23.0	0.95	0.75-1.21

PR: prevalence ratio; reference category - older adult individuals. 95%CI: 95% confidence interval. *Significant difference (p≤0.05) according to the chi-square test.

Among the main risk factors, those with the highest prevalence were physical inactivity (60.3%), depressive symptoms (56.7%), and hypertension (56.7%). Among the “other factors”, monolingualism (98.0%), visual deficit (84.7%), and irregular consumption of fruits (60.4%) and vegetables (53.5%) were more prevalent.

With respect to sex, the analysis showed a significant difference for the factors social isolation, depressive symptom, sleep disorder and irregular consumption of beans, which were more prevalent in women; and for alcohol use and regular consumption of soft drinks, which were higher in men. However, in the adjusted regression analysis, no differences were identified between sexes for any variable.

When comparing age groups, the older-aged group had a significantly higher prevalence of low education, hypertension, diabetes mellitus, and obesity. Adults had a higher prevalence of irregular consumption of beans and regular consumption of sweets and soft drinks. Adjusted regression analysis confirmed a significant difference for schooling, with older adult individuals having a higher prevalence of low education (PR=1.27; 95%CI 1.04-1.55).

## DISCUSSION

The risk factors for dementia that had the highest prevalence were physical inactivity, depressive symptoms and high blood pressure; among the “other factors”, they were monolingualism, visual deficit and poor diet. Regression analysis revealed an association between being an older adult and having low education.

A systematic review and meta-analysis of longitudinal cohort studies on physical activity and risk of dementia found that participants without dementia with high levels of physical activity had a 14% lower risk of having dementia when compared to participants without dementia with low levels of activity.^13^ Studies demonstrate that physical activity should be regular and of high intensity.^14^ ^,^ ^15^ However, it was not possible to determine the ideal duration and intensity of the activity to maximize the possible protective effects.^14^

The prevalence of depressive symptoms was 56.7%, which can be considered high in relation to studies in the Brazilian population.^16^ ^,^ ^17^ Depression is a social problem of increasing epidemiological relevance, present in all age groups. In the older population, depressive symptoms may be related to the onset of dementia, and it is not clear whether it acts as a risk factor for the development of a dementia process^2^ ^,^ ^16^ ^,^ ^17^ ^,^ ^18^ ^,^ ^19^ ^,^ ^20^ or as a prodrome of dementia.^21^ Individuals with a previous history of depression have approximately twice the risk of developing dementia than those without a history of depression, and when depression affects the older-aged people, this relationship tends to be stronger, and prolonged exposure to the neuroinflammatory effects of depression may trigger pathological mechanisms of dementia.^18^ On the other hand, in a cohort study that followed individuals for 28 years before the development of dementia, data did not provide support for the hypothesis that depressive symptoms increase the risk of dementia; the association observed between the two is possibly attributed to common causes or to the effects of preclinical dementia.^21^ Thus, further studies are needed to elucidate the biological mechanisms underlying this association.

Depression was more prevalent in women (62.4%), which is in line with the literature.^2^ ^,^ ^16^ ^,^ ^17^ ^,^ ^22^ It should be noted that men may be less willing to admit the occurrence of depressive symptoms and that, regardless of gender, a rigorous evaluation of these symptoms can help identify high risk profiles for dementia that may benefit from timely interventions.^23^

It was observed that the prevalence of hypertension in the total sample (56.7%) was higher than that found in other national studies.^24^ ^,^ ^25^ The high prevalence found in the older adults (71.9%) corroborates the finding of several population-based studies conducted in Brazil in this age group that showed prevalence ratios in older adults varying from 75.6^26^ to 85%.^27^ Furthermore, in south Brazil, older-aged people with hypertension were 168% more likely to develop dementia.^25^ Evidence suggests that antihypertensive drugs, especially renin-angiotensin system blockers and calcium channel blockers, may have an impact on the reduction of the incidence and progression of cognitive decline and dementia.^28^ However, the relationship between blood pressure and the brain is complex and can be influenced by several factors such as age, chronicity and use of antihypertensive medication.^25^

Regarding diabetes mellitus, data showed a higher prevalence in older adult individuals. In a meta-analysis, the relative risk of dementia for patients with diabetes was estimated at 1.46.^28^ A prospective health study found the risk of dementia to be twice as high among diabetic patients who experienced a severe hypoglycemic event compared to those who did not.^29^ Another prospective survey found glycemic spikes associated with poorer initial cognitive ability and accelerated cognitive decline.^30^

The prevalence of low schooling was significantly higher among older adults compared to adults. Low schooling among older people is a consolidated theme in the literature,^2^ ^,^ ^31^ ^,^ ^32^ being relevant, especially in developing countries, in which the number of older adults who have a low level of education is higher.^25^ A systematic review that assessed the relationship between educational level and aging-related cognitive impairment found that individuals with higher education commonly maintained higher cognitive scores, which can contribute to the prevention of cognitive decline. That data should be viewed with caution, as there is a scarcity of studies with individuals with lower schooling.^33^

It is also important to consider that the population's schooling has been increasing in Brazil. Data show that the average number of years of schooling for Brazilians over 25 years of age went from 8.9 in 2016 to 9.4 in 2019. The illiteracy rate in older adults went from 20.4 to 18.0% in the same period.^34^ Therefore, the identified difference regarding low schooling can be seen in a good perspective, as it may reflect generation differences. In this way, we must point out the theory of cognitive reserve, which says that experiences such as education, occupation, cognitive ability, and physical and social activity during individuals’ lifetime, combined with genetic factors, can enhance the cognitive process and allow them to cope better with brain changes.^35^

In the present study, monolingualism was the most prevalent risk factor for dementia. Studies published in several countries have shown the protective effect of bilingualism against the clinical symptoms of dementia, delaying its onset by 4-5 years.^36^ ^,^ ^37^ Bilingualism promotes cognitive reserve and cerebral reserve, and the combination of these two factors can provide a neural structure that explains the delayed onset of dementia.^38^ Although the literature presents substantial evidence that bilingualism is a protective factor for the development of dementia,^36^ not all studies observed these relationships.^39^ However, despite the increase in schooling in Brazil in recent years,^34^ the country still has high rates of illiteracy. This makes it impossible to intervene in this risk factor in the country at this time, and the focus should initially be on increasing the population's education and cognitive reserve.

Studies investigating visual impairment and dementia have repeatedly shown changes in the physiology of the nervous system and suggest that the prevalence of visual loss in people with dementia is higher than that in the older adult population without dementia.^40^ ^,^ ^41^ A longitudinal study found that moderate to severe visual impairment can be a predictor and probably a risk factor for dementia.^38^ Since the relationship between visual impairment and dementia is not fully understood, studies are needed to determine whether screening and treatment for vision loss can delay cognitive decline.^42^ ^,^ ^43^ Although low visual acuity is considered a risk factor for dementia,^40^ ^,^ ^41^ ^,^ ^43^ there is little longitudinal evidence examining this association.^41^

There is much question about the potential of dietary interventions to protect cognitive decline during aging.^44^ Observational studies have found lower rates of cognitive decline and a reduced risk of dementia in individuals who consume more vegetables and fruits.^45^ One explanation for the effects of nutrition on dementia may be that a poor-quality diet offers less nutrients and energy to the brain. It is plausible that the low intake of vegetables causes a lack of micronutrients, such as vitamins, minerals and fiber, ultimately causing a negative effect on cognition.^46^ Vegetables are a diverse food group, rich in B vitamins and antioxidants (vitamins A, C and E), and they therefore offer protection against cognitive decline,^1^ ^,^ ^47^ just as the consumption of grains, vegetables and fruits can have a positive impact on cognitive decline. On the other hand, the consumption of alcohol, sweets and soft drinks can have a negative influence.^1^ ^,^ ^47^ A healthy diet including foods with antioxidant and anti-inflammatory properties regulate the immune system and can alter neuroinflammatory events related to the progression of cognitive impairment.^44^

Eating habits can be a promising approach in preventing cognitive decline or delaying the progression of dementia.^4^ However, previous studies regarding the quality of the diet and cognitive impairment in older-aged people have shown inconsistent results.^46^ ^,^ ^47^ Some studies indicate that it is not possible to state whether adherence to a healthy eating habit really changes the risk of cognitive decline^48^ and suggest the need for additional studies of dietary intervention to investigate the causal associations between diet and Alzheimer’s disease.^44^ Other factors such as geographic areas, eating habits, instruments for assessing diet and cognition,^1^ and the methodological heterogeneity between the different studies^49^ can also influence the relationships found between variables.

The present study had the following limitations. The cross-sectional design does not allow establishing a cause and effect relationship between variables; data cannot be generalized, as they represent only a sample of individuals living in a Brazilian city that does not have full PHC coverage. The participants were recruited based on the recommendation of PHC teams, featuring a non-random sample. The self-reported aspect of the answers to questions about hypertension, diabetes mellitus, and visual and auditory impairments, despite being widely used in research, should be viewed with caution. Also, the variable use of alcohol should also be carefully analyzed, as the amount of alcohol used was not asked.

Thus, the development of new studies with larger samples, in other regions of Brazil and in other developing countries is recommended, as well as longitudinal assessments of individuals who present the risk factors.

The data found here contribute to the available knowledge about risk factors for dementia in middle- and older- aged individuals. In our sample, the most prevalent main factors were physical inactivity, depressive symptoms and high blood pressure. Among the “other factors”, monolingualism, visual impairment, and poor diet stood out. In regression analysis, no significant differences were identified between sexes. As for age groups, the older adults had significantly lower schooling.

The percentage reduction of each of the factors can contribute to a decrease in the prevalence of dementia syndrome in the future. Thus, investment in the prevention of each of these factors is important. Our results are innovative in the sense of showing which factors are more prevalent and may help in guiding interventions, especially in Brazil, where investment in public policies is incipient. Factors such as physical inactivity and poor diet are absolutely subject to interventions, as long as there are public policies and trained professionals to guide them. In addition, early identification and screening for depressive symptoms, high blood pressure and visual impairment can contribute substantially to reducing the prevalence of dementia. We also highlight that we did not identify differences between sexes, which shows that interventions should be aimed at both men and women in a similar way. With regard to the age group, interventions should also be similar, but considering the lower level of education of the older adults. The importance of investing in the educational system to enhance the education level of the population, especially of the older adults, is a high point.
